# Railway Infrastructure Classification and Instability Identification Using Sentinel-1 SAR and Laser Scanning Data

**DOI:** 10.3390/s20247108

**Published:** 2020-12-11

**Authors:** Ling Chang, Nikhil P. Sakpal, Sander Oude Elberink, Haoyu Wang

**Affiliations:** 1 Department of Earth Observation Science, Faculty of Geo-Information Science and Earth Observation, University of Twente, 7514 AE Enschede, The Netherlands; nikhil17.sakpal@gmail.com (N.P.S.); s.j.oudeelberink@utwente.nl (S.O.E.); 2 Fugro B.V., 3515 ET Utrecht, The Netherlands; ha.wang@fugro.com

**Keywords:** railway infrastructure, Sentinel-1, SAR, structural health, settlement

## Abstract

Satellite radar interferometry (InSAR) techniques have been successfully applied for structural health monitoring of line-infrastructure such as railway. Limited by meter-level spatial resolution of Sentinel-1 satellite radar (SAR) imagery and meter-level geolocation precision, it is still challenging to (1) categorize radar scatterers (e.g., persistent scatterers (PS)) and associate radar scatterers with actual objects along railways, and (2) identify unstable railway segments using InSAR Line of Sight (LOS) deformation time series from a single viewing geometry. In response to this, (1) we assess and improve the 3-D geolocation quality of Sentinel-1 derived PS using a 2-step method for PS 3-D geolocation improvement aided by laser scanning data; after geolocation improvement, we step-wisely classify railway infrastructure into rails, embankments and surroundings; (2) we recognize unstable rail segments by utilizing the (localized) differential settlement of rails in the normal direction (near vertical) which is yielded from the LOS deformation decomposition. We tested and evaluated the methods using 170 Sentinel-1a/b ascending data acquired between January 2017 and December 2019, over the Betuwe freight train track, in the Netherlands. The results show that 98% PS were associated with real objects with a significance level of 25%, the PS settlement measurements were generally in line with the in-situ track survey Rail Infrastructure aLignment Acquisition (RILA) measurements, and the standard deviations of the PS settlement measurements varied slightly with an average value of 6.16 mm.

## 1. Introduction

Satellite radar interferometry (InSAR) is a precise and efficient technique to map surface dynamics on Earth caused by natural process and anthropogenic activities [1,2,3,4,5]. Examples are using InSAR for monitoring surface deformation due to e.g., volcano eruption, earthquake, landslide, mining, and oil/gas extraction [6,7,8,9,10,11,12,13,14]. Thanks to the dramatic increase of high spatio-temporal resolution satellite radar (SAR) data, a recent development in the InSAR application has shifted the focus to monitoring individual civil infrastructure such as buildings, railways and bridges. References [15,16,17,18,19] have demonstrated the applicability of using Time-Series InSAR (TS-InSAR) such as PSI (Persistent Scatterer Interferometry) [20,21], for systematically monitoring line-infrastructure (which is expanded in one direction but has a limited scale in the other direction)—railways, dams, tunnels, and highways. These studies show that the detected coherent radar scatterers such as persistent scatterers (PS) along such line infrastructure have a comparable spatial observation density w.r.t. ground-instrument observations, and the precision of the corresponding (relative) deformations in satellite Line of Sight (LOS) direction can be up to millimeter levels. However, the geopositioning precision of coherent radar scatterers is rather poor in the order of meters [22]. Therefore, directly and accurately associating coherent radar scatterers with actual ground targets based on TS-InSAR derived geolocation estimates is almost impossible, especially for line infrastructure, and using medium-resolution SAR data.

In [22], a novel method was developed to improve the PS geopositioning precision in three dimensions by decoding the PS position in a 3-D voxel and associating the PS to a physical object with known 3-D geolocation. The association of the PS interprets deformation behavior at the sub-infrastructure level [22,23]. In recent years studies were conducted to determine the PS geopositioning precision for high resolution SAR data [24,25,26]. For instance, the improvement for PS geopositioning precision for X-band (TerraSAR-X) in [25] resulted in the order of centimeter for 2-D and in the order of decimeter for 3-D. Scaling down the geolocation uncertainty to subcentimeter level is feasible with a known 3-D position ground object, i.e., a passive corner transponder (CR). Reference [27] showed for medium resolution SAR data such as Sentinel-1 a/b, 2-D positioning could be improved, ranging between decimeter and couple of meters, with CRs. In addition, [28] showed that using laser scanning data (LiDAR, e.g., AHN3), the Radarsat-2 (Extra Fine mode) derived PS geolocation along a railway line can be improved. This study will further test and evaluate the Sentinel-1 derived PS geolocation improvement aided by laser scanning data.

As a promising medium-resolution SAR mission, Sentinel-1a/b [29] deliver radar imagery routinely on a weekly basis, which have been employed for structural health monitoring of line infrastructure [16,30,31,32,33]. Specifically for railway line infrastructure, it is composed of various sub-structures, including rail track, sleeper, embankment, catenary, platform, surrounding buildings. Every sub-structure depreciates once it was built [34] and may have some specific structural health issue. To detect the structural health problem per sub-structure, it is relevant to categorize all sub-structures and identify unstable and unhealthy sub-structure(s). However, the classification in terms of structural health situation and instability identification, is not a standard TS-InSAR output, thereby, a tailored post analysis is required.

The main goal of this work is to assess and improve the geolocation quality of Sentinel-1 derived PS and classify sub-structures to identify deforming railway structures. This paper is structured as follows: Section 1 presents the current status and challenges of using Sentinel-1 SAR data for railway infrastructure monitoring. Section 2 briefly describes the method adopted to associate PS with LiDAR point and approach to classifying PS along the track, and method to estimate and evaluate settlements of PS. In Section 3 the test area and data are specified. Section 4 addresses the results and discussion, followed by the conclusions in Section 5.

## 2. Method

This section first reviews the 2-step method for PS geolocation improvement aided by LiDAR data, see Section 2.1 and Section 2.2, and geo-located PS classification method in Section 2.3. The way to identify unstable rail segments along with (deformation) measurement quality, is discussed in Section 2.4.

### 2.1. 2-D Geolocation Estimation Improvement

**Figure 1:**
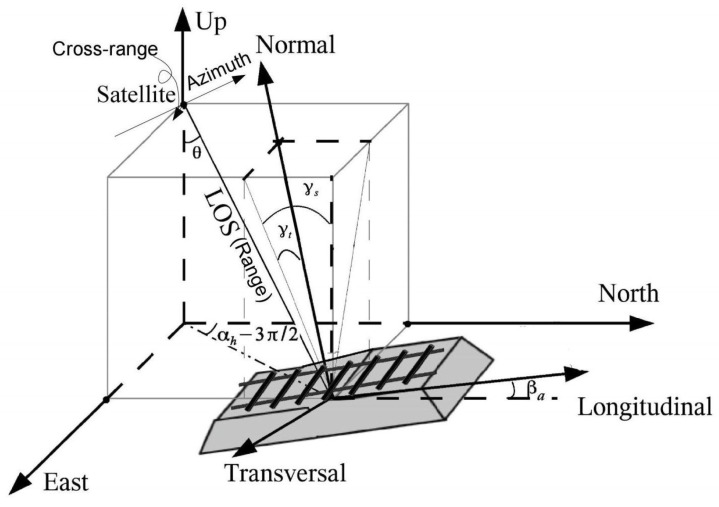
3D radar (with range, azimuth and cross-range directions), terrestrial reference (with east, north, and up directions), and local rail-track-fixed Cartesian coordinates (with transversal, longitudinal and normal direction). θ, $\gamma_{s}$, $\gamma_{t}$, $\alpha_{h}$, and $\beta_{a}$ separately represent the satellite look angle, longitudinal slope of track, cant of the track, satellite heading angle, and azimuth of the track w.r.t. the north. Adapted from [35].

The 2-step method for PS geolocation improvement starts with the PS geolocation estimation improvement in 2-D radar coordinates. The 2-D radar coordinates include the range and azimuth direction, which separately indicates radar signal direction and satellite flying direction, see Figure 1. Here, the PSI results, i.e., the PS along with deformation time series, are the input data for the PS geolocation improvement. The absolute 2-D positioning precision is dependent on the quality of the sub-pixel positioning, which is affected by systematic errors. Given a PS, denoted as *P*, its range and azimuth position, (${\underline{r}}_{P},{\underline{a}}_{P}$), are measured as [22]

(1) $${\underline{r}}_{P}=\frac{v_{0}}{2}\cdot \left({\underline{\tau }}_{0}+{\underline{\mu }}_{P}\cdot \underline{\Delta \tau }+\frac{2\cdot {\underline{r}}_{Q}}{v_{0}}-{\underline{\tau }}_{\mu_{Q}}\right)+{\underline{r}}_{{\mathrm{pd}}_{P}}+{\underline{r}}_{{\mathrm{tect}}_{P}}+{\underline{r}}_{{\mathrm{set}}_{P}},$$

(2) $${\underline{a}}_{P}={\underline{v}}_{s/c}\cdot \left({\underline{t}}_{0}+{\underline{\nu }}_{P}\cdot \underline{\Delta }t+\frac{{\underline{a}}_{Q}}{{\underline{\nu }}_{s/c}}-{\underline{t}}_{\nu_{Q}}\right)+{\underline{a}}_{{\mathrm{shift}}_{P}}+{\underline{a}}_{{\mathrm{tect}}_{P}}+{\underline{a}}_{{\mathrm{set}}_{P}},$$

where $v_{0}$ is the velocity of radar signal in vacuum [m/s], $\underline{\tau_{0}}$ is the time to the position of the first pixel, range sampling interval is $\underline{\Delta \tau }$, i.e., inverse of resampling frequency. The dimensionless pixel position in range direction is ${\underline{\mu }}_{P}$, ${\underline{r}}_{Q}$ and ${\underline{a}}_{Q}$ are empirically measured from the calibration target *Q*, ${\underline{\tau }}_{\mu_{Q}}$ and ${\underline{\tau }}_{\nu_{Q}}$ are measured in the commissioning phase, and the impact of second-order errors ${\underline{r}}_{{\mathrm{pd}}_{P}}$, ${\underline{r}}_{{\mathrm{tect}}_{P}}$, ${\underline{r}}_{{\mathrm{set}}_{P}}$ are path delays, tectonic delay, and SET (Solid Earth Tides) respectively. ${\underline{v}}_{s/c}$ denotes the satellite platform velocity along the orbit [m/s]. The first emitted pulse time of the scene is denoted as ${\underline{t}}_{0}$, the dimensionless pixel position in azimuth direction is ${\underline{\nu }}_{P}$, the inverse repetition frequency (PRF) is $\underline{\Delta t}$, the position shift of the pixel in the azimuth direction, ${\underline{a}}_{{\mathrm{shift}}_{P}},{\underline{a}}_{{\mathrm{tect}}_{P}},{\underline{a}}_{{\mathrm{set}}_{P}}$, are caused by the azimuth timing delay, tectonic, and SET respectively.

### 2.2. 3-D Geolocation Estimation Improvement

The second step of the 2-step PS geolocation improvement aims to improve the 3-D geolocation estimation with the aid of LiDAR. The 3-D radar coordinates include range, azimuth, and cross-range directions, in which the cross-range direction is orthogonal to the range-azimuth plane, see Figure 1. The position of PS, *P*, in the cross-range direction can be computed by [22]

(3) $${\underline{c}}_{P}={\underline{r}}_{P}\cdot {\underline{\theta }}_{PR},$$

where ${\underline{\theta }}_{PR}$ is the change in look angle θ. *R* indicates the reference (PS) point. Equation (3) implies that on improving 2-D positioning (${\underline{r}}_{P},{\underline{a}}_{P}$), the estimate in the cross-range direction can be adjusted.

#### 2.2.1. 3-D Error Ellipsoid Generation

**Figure 2:**
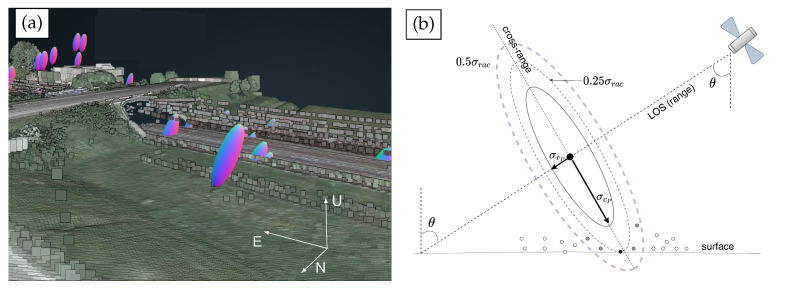
(**a**) Example of error ellipsoid from Potree viewer (center latitude and longitude is 51.840° N, 5.030° E). The ratio (azimuth: range: cross-range = 1:1.5:6) of semi-axis length is taken for visualization purposes. (**b**) A cross-section of error ellipsoid in range and cross-range dimensions. The error ellipsoid is centered at PS (${\underline{r}}_{P},{\underline{c}}_{P}$) in range and cross-range plane, with the semi- major and minor length $\sigma_{c_{P}}$ and $\sigma_{r_{P}}$. The associated real object on surface for this PS is denoted as the black solid circle given 25% significance level, when having the 3 × 3 error matrix $\sigma_{\mathrm{rac}}=\mathrm{diag}\left(\sigma_{r},\sigma_{a},\sigma_{c}\right)$. Adapted from [23].

After the (${\underline{r}}_{P},{\underline{a}}_{P},{\underline{c}}_{P},$) estimation in range, azimuth and cross-range directions using Equations (1)–(3), the improved 3-D PS geolocation estimation associated with its error ellipsoid is used to perform the geometric match with real objects. The 3-D error ellipsoid is structured by the (co)variance matrix in range, azimuth and cross-range directions, $Q_{\mathrm{rac}}$. Using a transformation matrix R, $Q_{\mathrm{rac}}$ can be projected to a terrestrial reference coordinates (with east, north, and up directions, see Figure 1), denoted as $Q_{ENU}$. $Q_{ENU}$ can be computed by [23,36],

(4) $$Q_{ENU}=RQ_{rac}R^{T}=R\left[\begin{array}{ccc} \sigma_{r_{P}}^{2} & \; & \\ \; & \sigma_{a_{P}}^{2} & \\ \; & \; & \sigma_{c_{P}}^{2} \end{array}\right]R^{T}=\left[\begin{array}{ccc} \sigma_{e}^{2} & \sigma_{en}^{2} & \sigma_{eu}^{2}\\ \sigma_{en}^{2} & \sigma_{n}^{2} & \sigma_{nu}^{2}\\ \sigma_{eu}^{2} & \sigma_{nu}^{2} & \sigma_{u}^{2} \end{array}\right],$$

where the variance in range, azimuth, and cross-range directions are described as $\sigma_{r_{P}}^{2}$, $\sigma_{a_{P}}^{2}$ and $\sigma_{c_{P}}^{2}$. $\left(\sigma_{e}^{2},\sigma_{n}^{2},\sigma_{u}^{2}\right)$, and $\left(\sigma_{eu}^{2},\sigma_{en}^{2},\sigma_{nu}^{2}\right)$, are the variances and covariances in east, north, and up directions. An error ellipsoid example from Potree viewer is displayed in Figure 2a. The ellipsoid has the longest semi-axis length in cross-range and up directions due to high uncertainty in the cross-range and up directions [22].

#### 2.2.2. Association with LiDAR Points

The accurate information (e.g., high-precision Digital Surface Model (DSM)) of the LiDAR point cloud on geo-objects, is treated as a (reference) real-object datum, cf. [15,22,35,37]. To compute the offset between PS geolocation with the error ellipsoids and LiDAR data, and geometrically associate PS to their most likely points in the LiDAR point cloud, the Nearest Neighbor Linking (NNL) approach [23] can be employed. The NNL approach is customized upon the K-Dimensional tree based Nearest Neighbor searching method which is widely used in point cloud data, to find the point in a given set that is closest in e.g., spatial distance to a point of interest [38,39,40,41]. Prior to conduct the Nearest Neighbor searching, the NNL approach requires the Whitening transform **W** to project both PS and LiDAR point cloud on the eignevectors of $Q_{ENU}$,

(5) $$W=E^{-1}D^{-\frac{1}{2}}E^{T},$$

where **E** and *D* indicate the eigenvalues and diagonal matrix of the eigenvectors of $Q_{ENU}$, respectively. In this regard, the Nearest Neighbors are the statistically nearest rather than the nearest in spatial distance, i.e., Euclidean metric distance. Figure 2b illustrates the graphic result of the NNL. For instance, for alleged $0.25\sigma_{\mathrm{rac}}$ case, known that error ellipsoid (built based on $\sigma_{\mathrm{rac}}=\mathrm{diag}\left(\sigma_{r},\sigma_{a},\sigma_{c}\right)$) is centered at PS (${\underline{r}}_{P},{\underline{c}}_{P}$) in range and cross-range plane, with the semi-major and minor length $\sigma_{c_{P}}$ and $\sigma_{r_{P}}$, and allowing 25% significance level, the associated real object on surface for this PS is detected and denoted as the black solid circle. The gray-colored and white-colored circles in the proximity located within/beyond 50% significance level are discarded.

### 2.3. Geo-Located PS Classification

To differentiate railway sub-structures from each other, and interpret the rail sub-structure’s deformation dynamics, the associated PS points are required to be classified spatially. With the 2-step method for PS geolocation improvement, all PS points are associated with the (LiDAR-built) real-world objects, and inherit the geolocation and feature attributes (including e.g., ground, buildings, water bodies, and vegetation labels) of the LiDAR point cloud. Such newly assigned attributes of PS are used for the geo-located PS classification.

Two main steps are proposed for categorizing geo-located PS: (1) coarse classification, PS are classified into ground, buildings, water bodies, and vegetation classes, using feature attributes; The PS on (left and right) rails and embankment are sorted in ground class. The PS on station platforms, surrounding buildings are sorted in building class. As PS are point-wise coherent scatterers, and thereby no PS is expected from vegetation areas if there is no artificial/natural ground object. We then label PS in water bodies and vegetation classes as erroneous geo-located PS. (2) fine classification, PS on rails are further differentiated from embankments using the mm-level-accuracy railway geometry measurements obtained by the in-situ railway geometry data. Particularly for Sentinel-1 mission, we have to define a spatial buffer such as 5 m, to fine-classify PS which originate from rails and embankment areas, separately. Note that Potree can be used to verify the PS classification, and it is useful for relatively small target recognition and validation, such as bridges.

### 2.4. Differential Settlement Detection

The railway instability can manifest itself as localized differential settlement of railway segments. The settlement is the deformation in the normal direction in rail-track-fixed Cartesian coordinates in the transversal, longitudinal and the complementing normal direction, see Figure 1. The transversal and longitudinal direction separately indicate cross-track and along-track direction of the rails, and the normal direction is near-vertical, and orthogonal to the transversal-longitudinal plane.

To obtain settlement measurements from the line-of-sight (LOS) deformations, the LOS deformation decomposition is applied, shown as [5],

(6) $$d_{\mathrm{LOS}}=p^{T}d_{\mathrm{geo}},$$

where $d_{\mathrm{LOS}}$ indicates the LOS deformation [mm], the projected 3-D deformation components in a terrestrial reference coordinates, are denoted as $d_{\mathrm{geo}}={\left[d_{e},d_{n},d_{u}\right]}^{T}$, *p* is the projection matrix, shown as $p={\left[-\sin\theta \cos\alpha_{h},\;\sin\theta \sin\alpha_{h},\;\cos\theta \right]}^{T}$. The satellite look angle and the heading are denoted as θ and $\alpha_{h}$.

Converting the east-north-up measurements $d_{\mathrm{geo}}$ into a local, track-fixed, Cartesian reference system is implemented by $d_{\mathrm{geo}}=R_{1}\;R_{2}\;R_{3}\;d_{\mathrm{track}}$, in which $d_{\mathrm{track}}={\left[d_{T},d_{L},d_{N}\right]}^{T}$ (with the components in the transversal, longitudinal and normal direction), and the three rotation matrices are [35]

(7) $$R_{1}=\left[\begin{array}{ccc} \cos\beta_{a} & \sin\beta_{a} & 0\\ -\sin\beta_{a} & \cos\beta_{a} & 0\\ 0 & 0 & 1 \end{array}\right],R_{2}=\left[\begin{array}{ccc} 1 & 0 & 0\\ 0 & \cos\gamma_{s} & -\sin\gamma_{s}\\ 0 & \sin\gamma_{s} & \cos\gamma_{s} \end{array}\right],$$

$$R_{3}=\left[\begin{array}{ccc} \cos\gamma_{t} & 0 & \sin\gamma_{t}\\ 0 & 1 & 0\\ -\sin\gamma_{t} & 0 & \cos\gamma_{t} \end{array}\right],$$

where $\beta_{a},\left[-90^{{}^{\circ}},+90^{{}^{\circ}}\right)$ is the azimuth of the track relative to the north, $\gamma_{s}$ and $\gamma_{t}$ represent the longitudinal slope of the track and the cant of the track respectively. To obtain a unique solution of the 3-D deformation components, LOS deformations from at least three different viewing geometry satellites are recommended. Otherwise, additional constraints should be introduced.

Assuming deformation is subtle in the transversal and longitudinal directions i.e., $d_{T}$ and $d_{L}$ are close to zero, $d_{\mathrm{LOS}}$ can be directly projected onto the normal direction, using

(8) $$d_{\mathrm{LOS}}=\left[\sin\theta \cos\alpha_{h}\sin\beta_{a}\sin\gamma_{s}-\sin\theta \sin\alpha_{h}\cos\beta_{a}\sin\gamma_{s}+\cos\theta \cos\gamma_{s}\right]d_{N}.$$

**Figure 3:**
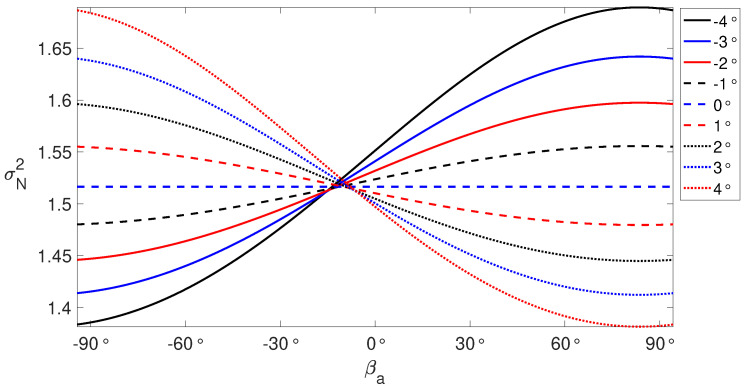
$\sigma_{N}^{2}$ values in response to the variation of $\beta_{a}$ [$-90^{{}^{\circ}},\;90^{{}^{\circ}}$] and $\gamma_{s}$ [$-4^{{}^{\circ}},\;4^{{}^{\circ}}$]. The Sentinel-1 satellite look angle $\theta =35.7^{{}^{\circ}}$ and heading $\alpha_{h}=349.8^{{}^{\circ}}$ are used.

According to the error propagation law, given the measurement uncertainty (i.e., variance) $\sigma_{\mathrm{LOS}}^{2}$ of $d_{\mathrm{LOS}}$, the measurement uncertainty $\sigma_{N}^{2}$ in the normal direction can be computed by

(9) $$\sigma_{N}^{2}={\left(A^{T}{\left(\sigma_{\mathrm{LOS}}^{2}\right)}^{-1}A\right)}^{-1},$$

where $A=[\sin\theta \cos\alpha_{h}\sin\beta_{a}\sin\gamma_{s}-\sin\theta \sin\alpha_{h}\cos\beta_{a}\sin\gamma_{s}+\cos\theta \cos\gamma_{s}]$. Assuming $\sigma_{\mathrm{LOS}}^{2}$ equals to 1 mm^2^, with the variation of $\beta_{a}$ between [$-90^{{}^{\circ}},\;90^{{}^{\circ}}$] and $\gamma_{r}$ between [$-4^{{}^{\circ}},\;4^{{}^{\circ}}$], the resultant $\sigma_{N}^{2}$ is illustrated in Figure 3 using Equation (9). When $\beta_{a}$ approaches to zero, implying the railway runs northwards, $\sigma_{N}^{2}$ is rather invariant with the variation of $\gamma_{s}$. Having larger values of both $\gamma_{s}$ and $\beta_{a}$, $\sigma_{N}^{2}$ is rather poorer. $\sigma_{N}^{2}$ goes better when having smaller values of both $\gamma_{s}$ and $\beta_{a}$.

The localized differential settlement can be computed by

(10) $$d_{N,(P,Q)}=d_{N,(P)}-d_{N,(Q)},$$

where $d_{N,(P)},d_{N,(Q)},d_{N,(P,Q)}$ represent the settlement of two (PS) adjacent point *P* and *Q* and the differential settlement between *P* and *Q*. The standard deviation of $d_{N,(P,Q)}$ is formulated as $\sigma_{N,(P,Q)}=\left(\sigma_{N,(P)}+\sigma_{N,(Q)}\right)$, ($\sigma_{N,(P)},\sigma_{N,(Q)}$ are the standard deviation of the settlement estimates of point *P* and *Q* respectively, cf. Equation (9)). For instance, having $\sigma_{N,(P)}=1.22$ [mm] and $\sigma_{N,(Q)}=1.25$ [mm], $\sigma_{N,(P,Q)}\approx 2.47$ [mm]. It implies that 1 mm LOS deformation error of point *P* and *Q* propagates itself to the localized differential settlement estimation of *P* and *Q* with 1.47 times larger. The unstable rail segments can be identified when the associated localized differential settlements exceed the predefined threshold.

## 3. Test Site and Data Description

**Figure 4:**
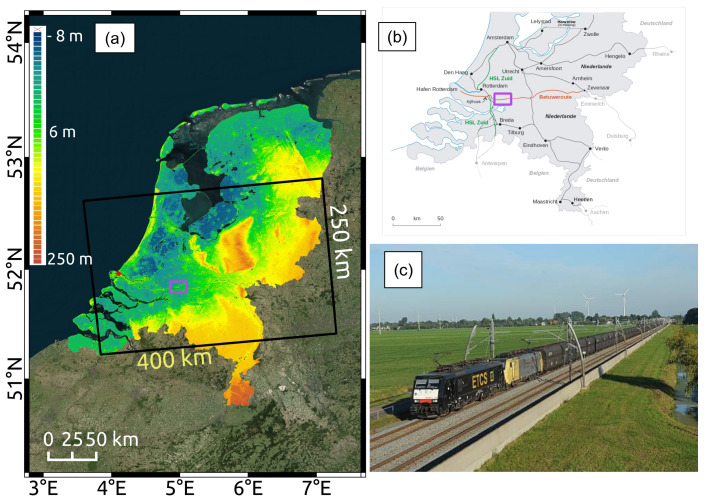
(**a**) Sentinel-1 satellite radar (SAR) data coverage (in black) over the Netherlands superimposed on an AHN3-derived Digital Surface Model (DSM) map. The extent of the test area is shown in purple. A GNSS station, as a reference point, is indicated in the red circle. (**b**) Dutch main railway network with the Betuwe route in red. (**c**) Train carrying heavy freight on the Betuwe dual track.

The test area selected for this study is a railway segment of *Betuwe Freight Corridor*. The dual-track rail corridor (153 km within the Netherlands, 172 km in total) was built to operate transport goods connecting Rotterdam port to central Europe and is important for the regional economy (see Figure 4b,c). A stretch of approx. 13 km long and a buffer area of 100 m of the corridor is monitored in this study (indicated by the magenta square in Figure 4a,b).

In this study, 170 Sentinel-1a/b (S-1a/b), SAR data in ascending orbit, in VV polarimetric channel, acquired between 4 January 2017 and 8 December 2019, were used. The S-1a/b spatial coverage is indicated in black in Figure 4a and the used SAR data details are specified in Table 1.

The coverage of AHN3 LiDAR is illustrated in Figure 4a and details are specified in Table 2. In comparison, three AHN3 point cloud tiles were processed to generate high-precision Digital Terrain Model (DTM) and Digital Surface Model (DSM) models for the test area shown in magenta, which was ∼96 km^2^.

## 4. Results and Discussion

**Figure 5:**
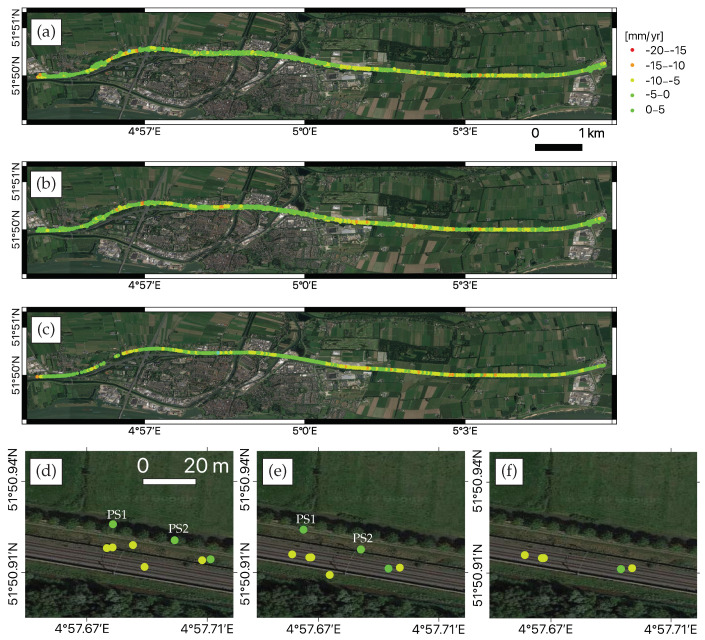
Line of Sight (LOS) deformation velocity map (**a**) before and (**b**) after 3-D geolocation improvement, and (**c**) of persistent scatterers (PS) on rails. Subfigures (**d**–**f**) separately illustrate the zoomed-in map before and after 3-D geolocation improvement, and of PS on rails, over an example area.

By applying a standard PSI approach, 170 S-1a/b SAR images were processed and the PSI results over the test area were generated. 3328 PS points were identified and unevenly distributed along the railway track, with a 100 m buffer zone at the both side of rails. The LOS deformation map prior to 3-D PS geolocation improvement is displayed in Figure 5a. The deformations of all PS were with respect to a common reference point which was a GNSS station located at 51.9667° N, 4.10° E, indicated in the red circle in Figure 4a. The LOS deformation velocity estimates mainly ranged between −10 and 5 mm/yr with an average deformation rate of −3 mm/yr. The average deformation velocity in the test area was −3.5 mm/yr, and 64% PS had a deformation velocity between [−5, +5] mm/yr.

### 4.1. PS Geo-Location Improvement and Association with Real Objects

**Figure 6:**
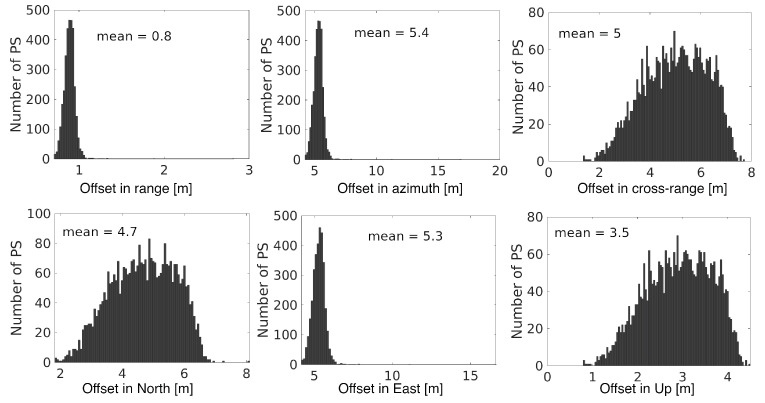
3-D geolocation offset estimates [m] in radar coordinates with range, azimuth and cross-range directions (top row), and in terrestrial reference coordinates with latitude, longitude and height (bottom row). The ’mean’ is the mean of the offset values.

To adjust the PS geolocation and improve positioning accuracy, we applied the 2-step method for geo-location improvement of those 3328 PS points, as mentioned in Section 2.1 and Section 2.2. First, we corrected the offset by removing systematic and second-order positioning errors to improve sub-pixel positioning, then further improved the cross-range estimation by employing the histogram match technique. The corrected position ($\underline{r},\underline{a},\underline{c}$) per PS along with its 3-D positioning uncertainty ($\sigma_{r}^{2},\sigma_{a}^{2},\sigma_{c}^{2}$) (e.g., with an averaged ellipsoid-axis-length ratio of 1:5:43) were used to construct an error ellipsoid, as explained in Section 2.2.1. To align PS and AHN3 LiDAR data in a common coordinate system, the 3-D PS geolocation estimates were transformed to the Dutch National Triangulation system (RD—‘Rijksdriehoeksstelsel’ in Dutch) and vertical (NAP—‘Normaal Amsterdams Peil’) reference system, denoted as *RDNAP* using an S-transformation [42], and then to WGS84. The estimated 3-D geolocation uncertainty in RDNAP was decorrelated using the whitening transform and then included to associate every PS point to the LiDAR point. The search for the nearest possible point with a successful association of 98% PS was achieved with a significance level of 25% (i.e., $0.25\sigma_{\mathrm{rac}}$ in Figure 2b). Note that lowering the significance level value led to the reduction of the association percentage. For instance, Ref. [28] obtained an association of 94% PS by customizing the significance level to 5%, for the high-resolution Radarsat-2 (Extra Fine mode) SAR data. At the end, 3259 PS out of 3328 were geometrically associated with the real objects. The correction in offset, of 3259 PS in radar and terrestrial reference coordinates, with the most likely points in LiDAR was completed by inheriting the positional attributes of LiDAR with PS, and introducing the geolocation uncertainty in each dimension, see Figure 6. The major offset correction was encountered in the azimuth and cross-range directions, whereas the offset correction in the range direction was relatively small. The PS geolocation estimates in the azimuth and cross-range directions were corrected in the order of several meters. The mean (absolute) height of 3259 PS was 0.5 m. The PS geolocation estimates in range were corrected from the order of decimeters to several meters. The LOS deformation velocity map after 3-D geolocation improvement is shown in Figure 5b. As an illustration, the zoomed-in map before and after 3-D geolocation improvement, over an example area, is shown in Figure 5d,e, respectively. For instance, the PS point PS1 which was originally and wrongly geolocated on a canal in Figure 5d, was re-geolocated at a trail area in Figure 5e. The PS point PS2 which was geolocated on a tree in Figure 5d, was re-geolocated and associated to the foot of a power pole in Figure 5e.

### 4.2. PS Classification Map

**Figure 7:**
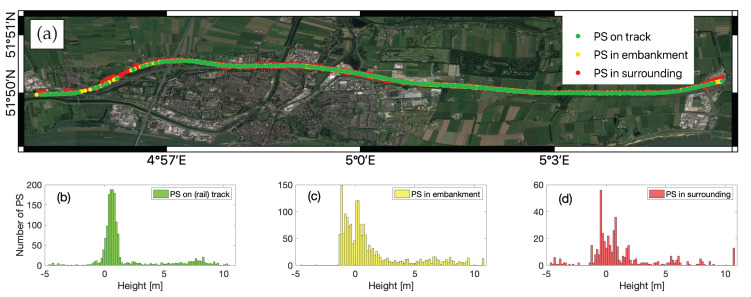
(**a**) Classification of PS associated to railway infrastructure. Height histogram of PS on track in green (**b**), PS in embankment in yellow (**c**), and PS in surrounding in red (**d**).

After the association of each PS point to the physical object, the PS points were classified into three classes in terms of their association within the proximity of railway track, based on the LiDAR-assigned feature attributes, railway geometry data, and PS height estimates (Section 2.3). For instance, ground-level PS points were categorized in terms of the feature attributes. Then we differentiated the PS associated to rails and embankments using railway geometry measurement (provided by Fugro) and PS height information. Considering the S-1a/b medium spatial resolution, we had to define the threshold of 10 m for rail track, between 10 m and 15 m for embankment area and above 15 m as surrounding area. 94% of the 3259 PS points were associated to the terrain, among which 1552 PS points were associated to rail track. Figure 5c shows the LOS deformation velocity map of PS on rails and Figure 5f depicts the zoomed-in map over an example area. Almost all of them had a [−5, 5] mm/yr deformation rate and the average deformation rate was −4 mm/yr. Figure 7a displays the PS classification map, with three classes: PS on rail track in green, PS in embankment in yellow, and PS in surrounding in red. Apart from 1552 PS on rail, 1030 PS points were associated within embankment zone and the remaining 677 PS points were associated with surrounding areas. The corrected PS height histogram is shown in Figure 7b. The mean height of PS associated with the rail track, within embankment, and in surroundings was 1.6 m, 1.2 m, and 1.2 m respectively.

### 4.3. Settlement Detection and Quality Description

**Figure 8:**
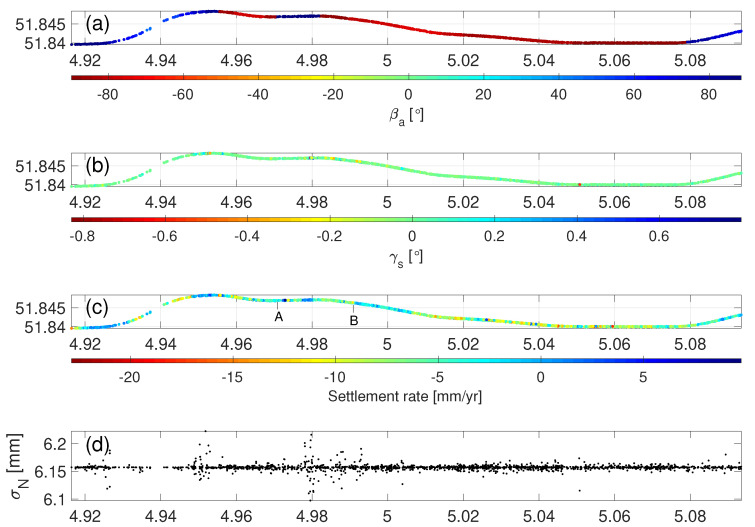
(**a**) The azimuth of track $\beta_{a}$, (**b**) longitudinal slope $\gamma_{s}$, (**c**) settlement rate in Normal direction, and (**d**) the standard deviation $\sigma_{N}$ in Normal when assuming $\sigma_{\mathrm{LOS}}=5$ mm, along the rails.

Using Equation (8), we projected the LOS deformation onto the normal direction, namely settlement. The deformation components in the longitudinal and transversal directions were assumed to be marginal (which was agreed and confirmed by the local rail asset manager). We computed the azimuth of track $\beta_{a}$ and longitudinal slope $\gamma_{s}$ of all 1552 PS, separately, see Figure 8a,b. As this rail mainly ran eastwards, the $\beta_{a}$ values of most of PS were close to either −90° or 90°. The $\gamma_{s}$ values ranged between −0.83° and 0.80°, and 98% PS points possessed the $\gamma_{s}$ values within [−0.2°,0.2°] which implies the rail tracks were laid on rather flat terrain. The corresponding settlement rate and associated standard deviation $\sigma_{N}$ (using Equation (9)), per PS, is presented in Figure 8c,d. Here we defined the standard deviation of LOS deformation measurements as 5 mm. The average value of $\sigma_{N}$ was 6.16 mm. The settlement measurement at a rail segment with a larger negative $\gamma_{s}$ value showed a bigger $\sigma_{N}$. For instance, the PS at 51.8483° N, 4.9518° E, having $\beta_{a}=75^{{}^{\circ}}$ and $\gamma_{a}=0.83^{{}^{\circ}}$, had a −2 mm/yr settlement rate associated with $\sigma_{N}=6.22$ mm.

### 4.4. Settlement Measurement Validation

**Figure 9:**
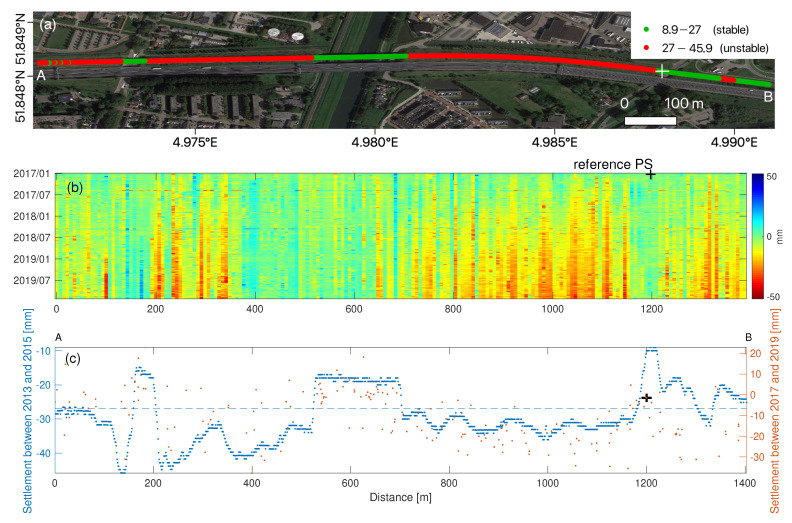
(**a**) Risk map over the segment A-B using the Rail Infrastructure aLignment Acquisition (RILA) data. The local reference PS point is indicated in the cross. (**b**) Cumulative settlement map for 196 PS points. (**c**) Settlement difference between January 2013 and February 2015 in blue and settlement difference between 4 January 2017 and 8 December 2019. The blue dashed line indicates the settlement threshold with the value of 27 mm. The location of the A-B segment is also indicated in Figure 8c.

In order to compare and validate the S-1a/b derived settlement with in-situ reference measurements, the measurements obtained by the train-borne measurement system Rail Infrastructure Alignment Acquisition (RILA) are referred to, cf. [43]. The RILA system integrates Global Navigation Satellite System (GNSS), Inertial Measurement Unit (IMU), LiDAR scanner, video cameras, and laser vision technologies to measure the absolute track geometry at line speed. The RILA survey campaign was conducted in January 2013 and February 2015 by Fugro, separately, with the measurement accuracy of 8 mm in the transversal and longitudinal, and 12 mm in the normal direction [44]. The absolute track geometry of an A-B segment with ∼1.4 km route length shown in Figure 9a was collected by the RILA system and used to compare and evaluate S-1a/b derived settlement. Per hectometer (100 m) there was a RILA measurement, in total there were 1410 RILA measurements over this segment. Considering the difference in data collection period of the RILA and S-1a/b measurements, we assumed that the settlement in this segment kept coherent since 2013, and no drastic change in settlement pattern occurred.

According to the criteria which are used to determine the stability of the track for trains with speed 70–100 km/h [45], a rail segment is labelled as stable when it retains structurally healthy condition with less than 27 mm settlement, while a rail segment is labelled as potentially unstable when it has larger-than-27 mm settlement. Therefore, using the settlement measurements per hectometer, between 2013 and 2015, shown in Figure 9c in blue, a binary riskmap was generated based on the criteria. Figure 9a shows the stable segments in green and unstable segments in red. Specifically, the rail segments on the fixed structure, i.e., bridges, ∼435 m route length, in green, behaved stably over time, thanks to the solid rail foundation. The rail segments on the soft soil, ∼975 m route length, in red, were subsiding, possibly due to soil compaction in response to e.g., heavy axle loads. The average settlement along the A-B segment is 30 mm, cf. [44].

There were 196 PS points over this A-B rail segment. We selected a local reference PS point with the coordinates 51.8467° N, 4.9878° E, −0.28 mm/yr in LOS. Using Equations (8) and (10), we generated the cumulative settlement w.r.t. the local reference PS point in the cross, see Figure 9b. The average standard deviation of the localized differential settlements, cf. $\sigma_{N,}\;\left(P,Q\right)$, was 12.3 mm, assuming $\sigma_{\mathrm{LOS}}=5$ mm. The cumulative settlement map shows the PS points on the fixed structure had marginal settlement, while PS points on the soft soil were moving downward, between 2017 and 2019. The settlement measurements derived by the RILA (in blue) and S-1a/b (in red), see Figure 9c, were matched in general. The measurement mismatching between RILA and S-1a/b could be caused by unexpected settlement pattern change between 2013–2015 and 2017–2019, and/or the rail maintenance.

### 4.5. Discussion

The approach to PS geolocation estimation improvement, as presented in Section 2.1 and Section 2.2, and demonstrated in Section 4.1 over the Betuwe freight train track, can facilitate the geometrical association of radar scatterers to real objects. As a generic approach, it can be employed over any area, and not limited to line-infrastructure, when satellite SAR and laser scanning data are both available. Yet, this approach does not take into account scattering mechanisms (mainly including surface, volume and dihedral scatterings). Hence, it does not guarantee the physical matching and association of radar scatterers and real objects. To realize physical matching and association, satellite SAR data in dual or quad polarization channels are recommended to be included, with which we can recognize scatterering mechanisms [46].

Regarding the geo-located PS classification method, addressed in Section 2.3 and demonstrated in Section 4.2, it allows us to differentiate PS on track from embankment and surrounding, by aggregating the feature attributes of PS, lidar and railway geometry data. Considering the medium spatial resolution of S-1a/b, a handful of PS could be possibly misclassified. To investigate the occurrence of the misclassified PS, classification after physical matching, as the follow-up research, could be of help.

Settlement detection method, as shown in Section 2.4 and tested in Section 4.3, enables us to reveal unstable railway segments with relatively large localized differential settlements, from satellite LOS deformation measurements. It is worth to note that when having satellite SAR data from more than two different viewing geometries, the deformation components in the longitudinal and transversal direction can be estimated in a better way, cf. [16]. Particularly for line-infrastructure, as it is unlikely having considerable non-elastic deformation in the longitudinal direction [47], the deformation component in the longitudinal direction can be considered as zero, and then we may use SAR data from merely two different viewing geometries to obtain the settlement and deformation component in the transversal direction. Besides, as we mentioned in Section 4.4, we compared and validated the detected settlement with RILA data. The S-1a/b measurements did coincide with RILA in general. However, we did also identify the measurement mismatching and hypothesize the cause. To further verify the cause, additional information on RILA and rail maintenance measurements acquired between 2017 and 2019 is needed, however, it is unavailable for our study.

## 5. Conclusions

This study demonstrated that InSAR is an unarguably powerful and promising technique to systematically monitor the structural health of line-infrastructure, railway infrastructure for our study, over large spatial- and temporal- scale, using medium-resolution Sentinel-1 SAR data. The laser scanning data, e.g., AHN3, can serve for the 3-D geolocation improvement and classification of all coherent radar scatterers, i.e., PS. Our results of the Betuwe case show that the estimates in east, north and up directions of all PS points were a couple of meters improved, using AHN3 data. With the improved geolocated PS population, the classification of PS was straight forward based on the position and features of the PS within rail structure premises, the number of PS points associated to the railways was increased by 14%, and the PS point density on rail was ∼2 PS points every 10 m^2^. With the help of the classification, the unstable rail segments which did experience considerably differential settlement could be easily and accurately identified and well interpreted. We expect that our proposed methods of classification and instability detection of railway infrastructure will add value to the early detection of rail fatigue for the rail asset managers, thereby improving the sustainable safety of rail transport.

## Figures and Tables

**Table 1:** Sentinel-1a/b data specification. az, rg are short for azimuth and range direction, respectively.

	Sentinel-1 a/b	Specification
	Pass	Ascending
	Track number	88
	Acquisition mode	Interferometric Wide swath (IW)
	Polarization mode	VV
	Swath width	250 km
	Imaging frequency	5.405 GHz
	Nr. of SAR images	170
	Spatial resolution	20 m × 5 m (az × rg)
	Temporal resolution	6 days
	Pixel spacing	13.94 × 2.32 (az × rg)

**Table 2:** AHN3 LiDAR data specification.

	AHN3 LiDAR	Specification
	Aqusition type	Airborne
	Imaging Frequency	800 nm and 1550 nm
	Height accuracy	5 cm × 5 cm
		(Systematic and Stochastic error)
	Planimetric accuracy	8 cm × 5 cm
		(Systematic and Stochastic error)
	Acquisition Mode	Laser scanned
	Point density	18 points per m^2^
	Pixel/Grid size	0.5 m × 0.5 m
	Total Nr. of tiles	3
	Temporal resolution	6 years
